# Enteric Coating Enhances the Biopharmaceutical Performance of a Silica–Lipid Formulation of Abiraterone Acetate

**DOI:** 10.3390/pharmaceutics17101289

**Published:** 2025-10-02

**Authors:** Ali Taheri, Ruba Almasri, Anthony Wignall, Felicia Feltrin, Kristen E. Bremmell, Paul Joyce, Clive A. Prestidge

**Affiliations:** Centre for Pharmaceutical Innovation, UniSA Clinical and Health Sciences, University of South Australia, Adelaide, SA 5000, Australia; ali.taheri@unisa.edu.au (A.T.); ruba.almasri@mymail.unisa.edu.au (R.A.); anthony.wignall@unisa.edu.au (A.W.); felfy002@mymail.unisa.edu.au (F.F.); kristen.bremmell@unisa.edu.au (K.E.B.); paul.joyce@unisa.edu.au (P.J.)

**Keywords:** mesoporous silica, lipid-based formulation, weakly basic drugs, in vitro lipolysis, in vivo pharmacokinetics, enteric coating

## Abstract

**Background/Objectives:** Lipid-based formulations are widely used to enhance the oral bioavailability of poorly water-soluble drugs. However, for weakly basic drugs with higher solubility under acidic conditions, precipitation and recrystallisation after gastric emptying can compromise a formulation’s ability to maintain the drug in a solubilised, absorbable state. To address this, we evaluated an enteric coating strategy to preserve the biopharmaceutical performance of a silica-solidified lipid-based formulation. **Methods and Results:** The model weakly basic BCS Class IV drug, abiraterone acetate, was loaded into a lipid-based formulation and solidified using mesoporous silica nanoparticles. In an in vitro lipolysis model, introducing the formulation only after the onset of the intestinal phase led to lower precipitation and over 50% greater drug presence in the aqueous phase compared to a two-stage gastric–intestinal digestion. In an in vivo pharmacokinetic study in Sprague Dawley rats, the silica–lipid formulation (6 mg/kg), delivered in gelatine minicapsules enteric-coated with Eudragit L100-55, resulted in a 2.6-fold higher systemic exposure compared to the non-coated formulation (*p* < 0.0001). **Conclusions:** These findings support the use of enteric coating for lipid-based formulations and silica nanoparticles containing weakly basic drugs as a strategy to maintain formulation integrity until reaching the small intestine.

## 1. Introduction

Lipid-based formulations are the most widely used nanomedicines in both clinical practice and development [1]. In addition to injectable forms, orally administered lipid-based formulations have been extensively researched and have progressed to clinical development and use [1,2]. As oral drug delivery systems, lipid-based formulations improve the bioavailability of poorly soluble drugs by facilitating solubilisation and dissolution through the generation of colloidal structures [3]. They present the drug in a non-crystalline, molecularly dispersed form, thereby overcoming the barrier posed by the crystal lattice, while the surfactants and lipids within the formulation help to maintain solubilisation and stabilise supersaturation [4,5]. Alongside lipid-based nanoparticles, inorganic nanoparticles, particularly mesoporous silica nanoparticles, are gaining prominence as drug delivery carriers, with several candidates advancing into late-stage clinical evaluation [6]. The large surface area of mesoporous silica nanoparticles makes them ideal vehicles for drug delivery systems, offering high loading capacity and tuneable release characteristics [7,8]. Interactions between drug molecules and the silica surface help to maintain dispersion of the drug at the molecular level, while the small pore size of mesoporous silica nanoparticles restricts nucleation and crystal growth [9,10].

Solidification can address significant challenges associated with liquid formulations, including limited stability, costly distribution, and storage conditions. It also facilitates precise dosing and improves patient adherence [11]. We have previously employed mesoporous silica to stabilise and enhance the performance of various liquid lipid-based oral formulations for poorly water-soluble drugs. This approach has resulted in improved in vitro solubilisation [12,13,14], enhanced pharmacokinetics in rodents [15,16,17], and promising safety and tolerability in clinical studies [18,19], confirming the potential of silica-stabilised lipid-based formulations to enhance the bioavailability of poorly water-soluble drugs. Upon administration of the solidified formulation, lipid structures disperse in the gastrointestinal media, forming colloidal structures that aid drug solubilisation. Meanwhile, the silica particles help delay drug nucleation and crystallisation, reducing precipitation and improving biopharmaceutical performance [20].

Abiraterone acetate, a Biopharmaceutics Classification System (BCS) Class IV compound, is an orally administered prodrug used for the treatment of metastatic prostate cancer. After conversion to its active metabolite, abiraterone, it inhibits cytochrome P450 17A1 (CYP17A1), an enzyme essential for androgen biosynthesis [21]. Various lipid-based formulations [22,23,24] and their combinations with silica nanoparticles [13,17,25] have been developed for abiraterone acetate. As a weakly basic drug, abiraterone acetate exhibits higher solubility at lower pH levels [21]. Consequently, in the gastric environment, there is a risk of the drug partitioning out of its delivery carrier. During the phase transition from gastric to the higher pH values in the intestinal compartment, rapid crystallisation and precipitation can occur, which is unfavourable for drug absorption and bioavailability [3,26].

Enteric coatings are widely employed to modify drug release from oral dosage forms, protect the formulation from the gastric environment, or mitigate gastric adverse effects [27]. This study aimed to investigate the effect of enteric coating on the performance of a lipid-based formulation confined within mesoporous silica nanoparticles for abiraterone acetate, a weakly basic model drug. It was hypothesised that the enteric coating would prevent early prodrug release in the gastric pH environment. This, in turn, would help preserve the solubilising benefits of the drug delivery system by gradually releasing the drug at the absorption site in the intestinal compartment, where both abiraterone and abiraterone acetate are less soluble due to the higher pH. This approach could reduce the rate of supersaturation upon gastric emptying, thereby decreasing crystallisation and variability in drug absorption caused by varying gastrointestinal conditions and prandial states.

## 2. Materials and Methods

### 2.1. Materials

Abiraterone acetate and abiraterone were sourced from Hangzhou Dayangchem Co. Ltd. (Hangzhou, China). The lipids Capmul MCM (caprylic and capric mono- and diglycerides) and Captex 300 (caprylic and capric triglycerides) were provided by Abitec (Columbus, OH, USA). Parteck SLC 500 silica was supplied by Merck Millipore (Bayswater, Australia). Syloid 244 FP silica was obtained from Grace Davison Discovery Sciences (Rowville, Australia). Isopropanol, ethanol, and acetonitrile were purchased from ChemSupply (Gillman, Australia). Pancreatin, Cremophore RH-40 (PEG-40 hydrogenated castor oil), Pluronic F127 (Poloxamer 407), phenacetin, sodium hydroxide pellets, hydrochloric acid, and formic acid were purchased from Sigma-Aldrich (Castle Hill, Australia). Eudragit L100-55 was obtained from Evonik Operations GmbH (Essen, Germany). Heparin sodium was purchased from Lyppard Australia (Beverley, Australia). High-purity Milli-Q water (Merck Millipore, Bayswater, Australia) was used throughout the study.

### 2.2. Formulation Preparation

A mixture of Cremophor RH 40 (35.0% *w*/*w*), Captex 300 (27.5% *w*/*w*), and Capmul MCM (27.5% *w*/*w*) was heated to 50 °C and vortex mixed for 10 s. Once the mixture cooled to room temperature, ethanol (10.0% *w*/*w*) was added, and the solution was vortex mixed for an additional 10 s. The equilibrium solubility of abiraterone acetate in this liquid vehicle, previously determined as 68.0 ± 7.0 mg/g [22], was used to load the drug at 80.0% of its solubility. Silica (Parteck SLC 500 or Syloid 244 FP) was blended with the liquid formulation at a 1:1 weight ratio relative to the vehicle to prepare silica-stabilised formulations.

### 2.3. Particle Characterisation

The particle size and polydispersity index (PDI) of the liquid formulation were determined using multi-angle dynamic light scattering (MADLS) (Zetasizer Ultra, Malvern Panalytical) following a 100-fold dilution in water. The zeta potential of the formulations was determined using electrophoretic light scattering (ELS) (Zetasizer Ultra, Malvern Panalytical) at neutral pH. Particle size and dispersibility of the solid formulations were assessed by laser diffraction (Mastersizer 3000, Malvern Panalytical) over 15 min after adding the silica–lipid powders (refractive index, 1.46) to water (refractive index, 1.33). All measurements were performed in triplicate.

### 2.4. In Vitro Gastrointestinal Lipolysis

A two-step, one-compartment gastrointestinal lipolysis model was employed. Fasted State Simulated Gastric Fluid (FaSSGF) and Fasted State Simulated Intestinal Fluid (FaSSIF) were prepared according to the manufacturer’s instructions (Biorelevant.com Ltd., London, United Kingdom). To prepare the intestinal enzyme, 1.2 g of porcine pancreatin was dispersed in 6 mL of FaSSIF, vortex mixed for 10 s, and centrifuged at 2268× *g* for 20 min at 4 °C. The resulting supernatant was collected and used for the experiments. For non-enteric-coated formulations, an equivalent of 3 mg abiraterone acetate was added to 10 mL of FaSSGF media, followed by the addition of 100 µL of gastric enzyme (Candida lipase, equivalent to 600 tributyrin units of lipase activity) after one minute. After a 30-min gastric phase, 18 mL of FaSSIF media was introduced to the vessel, and 2 mL of the intestinal enzyme was added after one minute, continuing the intestinal phase for 60 min. To simulate the impact of enteric coating, the silica–lipid formulation was introduced only after the FaSSIF medium was added to the vessel. Throughout the experiments, the lipolysis medium was continuously stirred and maintained at 37 °C in a thermostated glass vessel. The pH was controlled at 1.6 during the gastric phase and 6.5 during the intestinal phase using a pH-stat apparatus (902 Titrando, Metrohm, Switzerland) connected to a dosing system (Dosino 800, Metrohm, Switzerland), which contained 0.6 M sodium hydroxide. Aliquots (1 mL) were withdrawn from the lipolysis medium to measure the concentration of solubilised abiraterone acetate and abiraterone in the aqueous phase. Samples were collected at 1, 15, and 30 min during gastric lipolysis, and at 1, 5, 10, 15, 30, 45, and 60 min during intestinal lipolysis. Each aliquot was transferred into centrifuge tubes containing 10 µL of 0.5 M 4-BBA to inhibit lipase activity. The samples were then centrifuged at 12,470× *g* for 10 min to separate the precipitate from the clear supernatant. The supernatant was subsequently diluted with acetonitrile and spiked with an internal standard for LCMS analysis. All experiments were conducted in triplicate.

### 2.5. In Vivo Rat Pharmacokinetic Study

All rat experiments were approved by the South Australian Animal Ethics Committee (approval number U30-21) and conducted in accordance with the ARRIVE guidelines. The study adhered to the Principles of Laboratory Animal Care (NIH publication #85-23, revised 1985) and the National Health and Medical Research Council (NHMRC) Australian code for the care and use of animals for scientific purposes (8th edition, 2013). Pharmacokinetic studies were performed on male Sprague-Dawley rats (390–411 g; *n* = 4 per group), sourced from the Animal Resources Centre (Canning Vale, Australia). Rats were acclimatised for at least 5 days prior to any procedures. They were housed in pairs, with ad libitum access to water, and fasted overnight (~15 h) before dosing. Food was reintroduced 2 h post-dosing. For each animal, a dose equivalent to 6 mg/kg of abiraterone acetate was calculated based on pre-fasting body weight, and the corresponding amount of the silica–lipid formulation was loaded into size 9 gelatine capsules (Torpac Inc., Fairfield, NJ, USA). The enteric coating solution comprised 4% (*w*/*w*) Eudragit L100-55, 38% (*w*/*w*) isopropyl alcohol, 57% (*w*/*w*) acetone, and 1% (*w*/*w*) propylene glycol. Pre-filled capsules were placed in a holder with the body side down, dip-coated, and dried for 30 min. The holder was then inverted to position the capsule caps for coating, which were similarly coated and dried. This process was repeated to apply two enteric coating layers per capsule. Capsules were orally administered to rats using an intragastric dosing tube with 200 µL of water to facilitate dosing. Blood samples (200 µL) were collected from the saphenous vein at 20 and 40 min, and at 1, 1.5, 2, 4, 6, 8, and 24 h post-dose into heparinised tubes. Plasma was separated by centrifugation (21,000× *g*, 5 min, room temperature) and stored at −80 °C until analysis. At the conclusion of the study, rats were humanely euthanised by CO_2_ asphyxiation.

### 2.6. Liquid Chromatography-Mass Spectrometry

The analysis was performed using a Shimadzu LCMS 8030 system (Kyoto, Japan). A 1 µL sample aliquot was injected onto a Kinetex C18 column (50 × 3 mm, 2.6 μm particle size) from Phenomenex (Torrance, CA, USA), maintained at 40 °C. Analyte separation was achieved using a gradient elution protocol with mobile phase A (0.1% formic acid in water) and mobile phase B (0.1% formic acid in acetonitrile), delivered at a flow rate of 0.4 mL/min over 5.1 min. The gradient program was as follows: mobile phase B at 20% (0–0.5 min), 80% (3.5–4.5 min), and 20% (4.6–5.1 min). Mass transitions for abiraterone acetate, abiraterone, and phenacetin (internal standard) were m/z 392.2/332.25, m/z 350.1/156.15, and m/z 180.0/110.1, respectively. Ion detection was performed in multiple reaction monitoring mode with positive ionisation. Data processing was carried out using Lab Solutions software (Shimadzu, Kyoto, Japan). The lower limit of quantification (LLOQ) for the assay was 1 ng/mL.

### 2.7. Pharmacokinetic Analysis

Non-compartmental pharmacokinetic analysis was conducted using Aplos NCA (Aplos Analytics Inc., Orem, UT, USA). The area under the curve (AUC) was determined using the linear trapezoidal method. C_max_ and time to C_max_ (t_max_) were directly obtained from the individual plasma concentration-time profiles. Relative bioavailability (F_rel_) was calculated as the dose-normalised ratio of AUC_test_ to AUC_reference_.

### 2.8. Statistical Analysis

Data are presented as median [min, max] or mean (standard deviation, SD). All descriptive statistics, statistical tests, and plots were generated using GraphPad Prism 10.3.1 (GraphPad Software Inc., Boston, MA, USA). One way ANOVA followed by Tukey’s post-hoc test was used to compare study groups, with statistical significance set at *p* < 0.05.

## 3. Results and Discussion

### 3.1. Formulation Characteristics and Particle Size Analysis

Silica-stabilised lipid-based formulations of abiraterone acetate were obtained by combining the drug-loaded liquid formulation with Parteck SLC 500 or Syloid 244 FP. The liquid formulation had a drug loading of 5.2% *w*/*w*, and adding silica at a weight equivalent to the lipid-based vehicle yielded a free-flowing powder with a final drug loading of 2.6% *w*/*w*.

**Figure 1:**
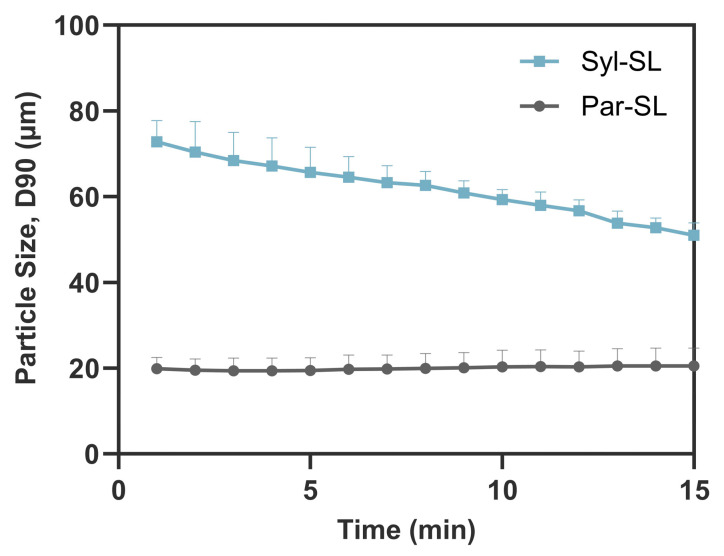
Particle size (D90) of Syloid silica–lipid (Syl-SL) and Parteck silica–lipid (Par-SL) formulations of abiraterone acetate during dispersion in water over time (mean ± SD, n = 3). D90 represents the particle diameter where 90% of the total sample volume is at or below that size.

Particle size analysis of the liquid formulation showed a monodisperse droplet size distribution of 30.4 ± 0.2 nm (PDI, 0.05). The zeta potential of the liquid formulation was slightly negative (−6.4 ± 1.5 mV), whereas the silica–lipid systems were more negatively charged (−32.6 ± 1.2 mV for Syloid silica–lipid [Syl-SL] and −18.7 ± 1.4 mV for Parteck silica–lipid [Par-SL]). Laser diffraction analysis of the dry powders showed that pure Syloid had a smaller median particle size (D50, 4.7 ± 0.5 μm) than pure Parteck (D50, 11.7 ± 0.1 μm). Consistent with these observations, the Syl-SL formulation had a smaller median particle size (D50, 8.0 ± 0.6 µm) than the Par-SL formulation (D50, 12.4 ± 1.5 µm) over 15 min. The Par-SL powders, however, exhibited superior dispersibility, maintaining a consistent size distribution span throughout the dispersion period, whereas the Syl-SL powders dispersed more slowly, with 10% of particles still exceeding 51.0 ± 2.9 µm at 15 min (Figure 1). The broader particle size distribution of the Syl-SL formulation, driven by the persistence of larger particles throughout the experiment, may impact dissolution behaviour and indicate a less stable system compared with the Par-SL formulation.

### 3.2. In Vitro Gastrointestinal Lipolysis

**Figure 2:**
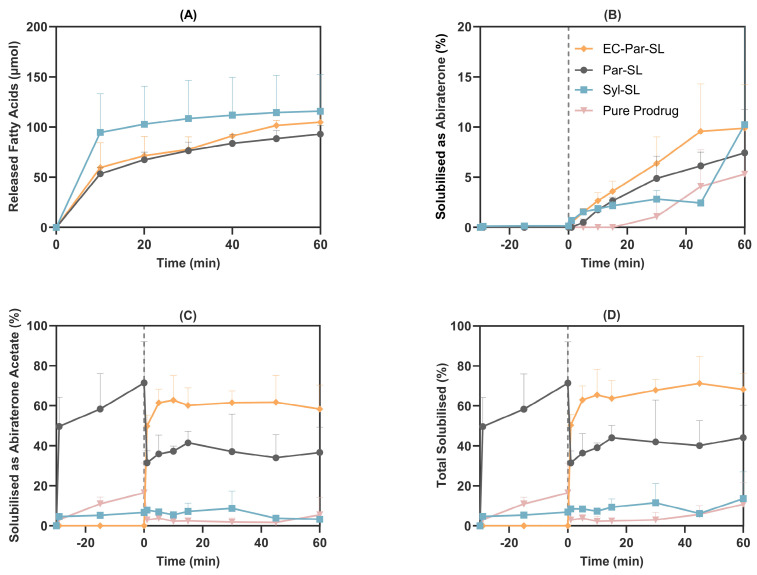
Two-step in vitro lipolysis of a 3 mg abiraterone acetate dose in different formulations. (**A**) Fatty acid release during intestinal lipolysis (datapoints shown every 10 min). Solubilisation profiles of abiraterone (**B**), abiraterone acetate (**C**), and total abiraterone species (**D**) in the aqueous phase under biorelevant fasted digesting conditions (mean ± SD, n = 3). The vertical dotted line represents the transition from gastric to intestinal media. Data for pure prodrug are adopted from a previous study [22].

Distinct digestion profiles were observed between the Syloid– and Parteck–lipid formulations. The Syloid–lipid formulation demonstrated rapid lipid digestion, marked by a faster onset and a greater extent of early fatty acid release. The Parteck–lipid formulation exhibited a more gradual digestion profile, with comparable levels of fatty acid release in the intestinal phase regardless of prior exposure to gastric conditions (Figure 2A). In hydrophilic silica nanoparticles, lipid adsorption into the pores increases surface area and, together with electrostatic repulsion between negatively charged fatty acids and the silica surface, facilitates lipase access to undigested lipids. The surface chemistry of silica can further modulate the digestion rate by influencing lipase interactions and the orientation of lipids within the pores [28,29,30,31].

Abiraterone acetate, a weakly basic compound, exhibited a pH-dependent solubilisation profile, with a tendency to precipitate upon transitioning from the acidic gastric phase to the more neutral pH of the intestinal phase [22]. In the FaSSGF, the pure prodrug showed moderate release, with 16.5 ± 14.9% of the dose dissolved after 30 min. Upon the addition of FaSSIF, the concentration of abiraterone acetate rapidly decreased, and after 1 min, only 2.9 ± 0.4% of the dose remained in the aqueous phase. After 60 min in the intestinal phase, some redispersion of the prodrug and hydrolysis to abiraterone occurred, reaching average concentrations of 14.0 ± 22.4 µM (5.5 ± 8.8% release) for abiraterone acetate and 13.5 ± 12.3 µM (5.3 ± 4.8% release) for abiraterone by the end of the experiment.

Solubilisation of abiraterone acetate by the Syl-SL formulation was poor, with less than 15% of the dose remaining in solution throughout the experiment, and the Syloid-based formulation was therefore not taken forward for further development. In contrast, the Par-SL formulation improved concentration of abiraterone acetate in the aqueous phase, reaching 547.1 ± 157.8 µM after 30 min in the gastric phase, corresponding to approximately 71.4 ± 20.6% of the total dose. As observed with the pure prodrug, addition of FaSSIF caused a rapid decrease in abiraterone acetate concentration, with approximately 31.5 ± 5.9% of the total dose remaining in the aqueous phase after 1 min. During the intestinal phase, some redissolution and prodrug hydrolysis occurred, resulting in 93.7 ± 32.3 µM (36.7 ± 12.6% release) of abiraterone acetate and 19.0 ± 11.0 µM (7.4 ± 4.3% release) of abiraterone by 60 min (Figure 2B–D).

To evaluate the impact of preventing abiraterone acetate release from the Par-SL system under acidic conditions, aqueous phase concentrations were measured when the formulation was dosed only after the phase transition to intestinal biorelevant media (EC-Par-SL). In this scenario, Par-SL rapidly dispersed in FaSSIF, resulting in 50.3 ± 5.3% of the dosed drug (128.6 ± 13.7 µM total) in the aqueous phase after just 1 min. This increased to 68.2 ± 7.9% (148.9 ± 30.7 µM of abiraterone acetate and 25.3 ± 11.1 µM abiraterone) in solution after 60 min (Figure 2B–D). Precipitation and recrystallisation of drug molecules can lead to low and variable oral bioavailability, a limitation that the in vitro lipolysis model suggests may be mitigated by bypassing gastric release through an enteric coating strategy.

The Parteck silica–lipid formulation effectively inhibited the rapid hydrolysis of abiraterone acetate. In the EC-Par-SL vessels, the average intestinal AUC_1–60 min_ of abiraterone acetate (152.2 ± 22.0 µM·h) was 9.7-fold higher than that of abiraterone (15.8 ± 6.6 µM·h). Similarly, in the Par-SL vessels, the average intestinal AUC_1–60 min_ values of abiraterone acetate (92.3 ± 25.6 µM·h) and abiraterone (11.0 ± 2.6 µM·h) differed by 8.4-fold. In contrast, with the pure prodrug, comparable AUCs were observed for abiraterone acetate (6.5 ± 6.9 µM·h) and abiraterone (5.0 ± 4.3 µM·h). Hydrolysis of abiraterone acetate in the intraluminal environment generates abiraterone supersaturation, which serves as a driving force for intestinal absorption [32]. However, a rapid onset of abiraterone supersaturation may promote precipitation and crystallisation in the proximal small intestine, potentially limiting absorption in more distal segments [33,34]. Therefore, limiting the rate of abiraterone acetate hydrolysis and moderating the rate of abiraterone supersaturation formation may enhance biopharmaceutical performance.

**Figure 3:**
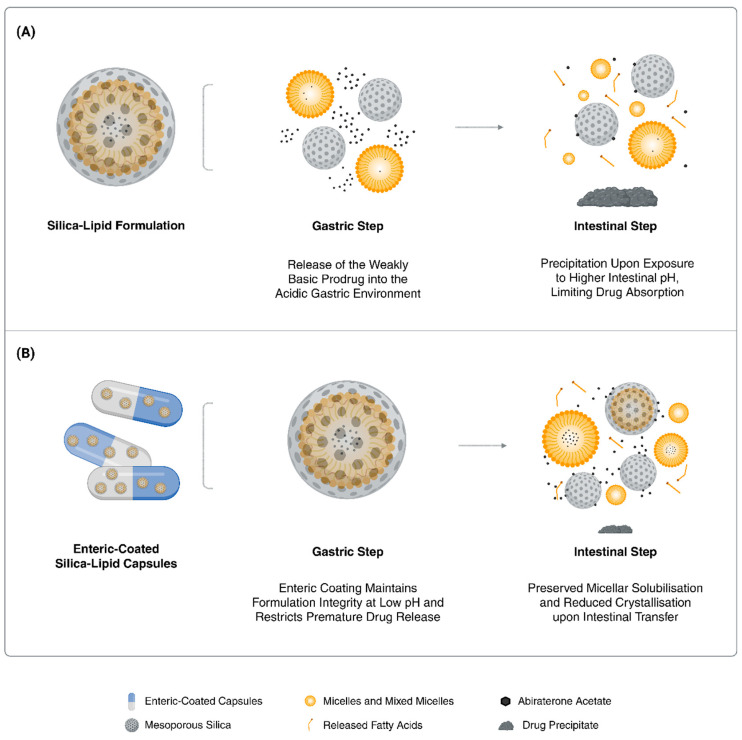
Schematic illustrating the structure and biopharmaceutical mechanism of an enteric-coated silica–lipid formulation of abiraterone acetate. (**A**) A silica–lipid formulation without enteric coating releases abiraterone acetate in the gastric environment, where high solubility at low pH is followed by rapid precipitation upon transition to intestinal pH, thereby limiting absorption. (**B**) An enteric-coated silica–lipid formulation prevents gastric release and preserves formulation integrity until reaching the intestine, where it sustains solubilisation, reduces precipitation, and enhances bioavailability.

Based on these observations, we proceeded to evaluate the enteric-coated Parteck silica–lipid abiraterone acetate (EC-Par-SL) formulation in vivo. The capsules were coated with EUDRAGIT L 100-55, an enteric polymer that ensures dispersion and dissolution starts only at a pH above 5.5, thereby protecting the formulation from early drug release in the gastric environment [35]. This approach maintains the integrity of the prodrug and the nanomedicine formulation until reaching the small intestine, the primary site of drug absorption (Figure 3).

### 3.3. In Vivo Rat Pharmacokinetics

**Figure 4:**
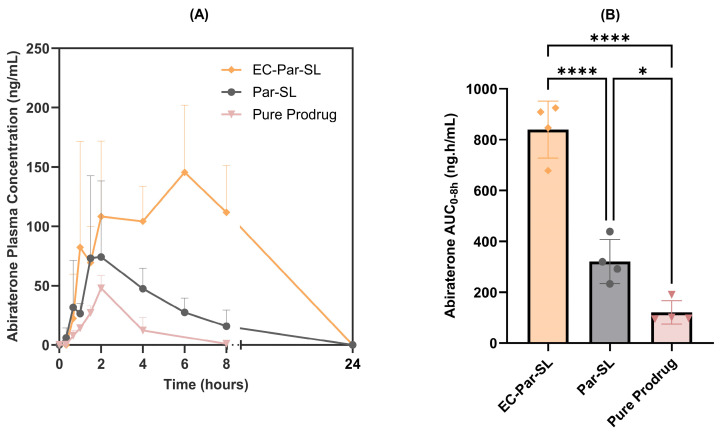
Plasma concentration-time profiles (**A**) and corresponding area under the curve (AUC) values (**B**) of abiraterone following oral administration of abiraterone acetate formulations in fasted male Sprague-Dawley rats: EC-Par-SL (6 mg/kg), Par-SL (6 mg/kg), and pure prodrug (25 mg/kg). Data for pure prodrug are adopted from a previous study [25]. Data are presented as mean and SD (n = 4). * *p* < 0.05, **** *p* < 0.0001.

The concentration-time profiles of abiraterone in fasted male Sprague-Dawley rats (Figure 4) and corresponding pharmacokinetic parameters (Table 1) show that enteric-coating the silica–lipid oral formulation improves systemic exposure to abiraterone by 28.7-fold (*p* < 0.0001) compared to pure abiraterone acetate and by 2.6-fold (*p* < 0.0001) compared to the non-coated silica–lipid formulation.

Enteric coating has been explored as a formulation strategy for poorly soluble, weakly basic drugs to limit drug release in the acidic gastric environment and thereby reduce the risk of crystallisation as the pH increases following gastric emptying. For instance, amorphous solid dispersions (ASDs) of delamanid, a weakly basic drug used in tuberculosis, showed >80% drug release in single-stage dissolution experiments at intestinal pH; however, rapid crystallisation and reduced release were observed in two-stage pH-shift studies [36,37]. This compromised release performance was addressed by applying an enteric coating (Acryl-EZE II) to the ASD tablets, which improved performance and reduced variability across different media and pH conditions [37]. Similarly, Eudragit L100 coating of an ASD formulation of nintedanib, a weakly basic drug used in idiopathic pulmonary fibrosis, mitigated drug crystallisation in two-stage dissolution studies and enhanced oral absorption and bioavailability in vivo [38]. In another example, an enterically coated complex of abiraterone acetate with Soluplus significantly improved fasted-state bioavailability and eliminated food-effect variability in both preclinical and clinical pharmacokinetic studies [39].

Enteric coating has been applied to lipid-based formulations [40,41,42,43] and mesoporous silica nanoparticles [44] to protect against chemical instability under gastric conditions and to minimise gastric and oesophageal irritation. Our findings demonstrate the utility of an enteric coating strategy in enhancing the performance of an enabling formulation for a poorly soluble, weakly basic drug. A two-stage biorelevant lipolysis experiment using the silica–lipid formulation revealed considerable drug precipitation as the pH shifted from gastric to intestinal conditions, whereas a single-stage study indicated higher extent of supersaturation under intestinal conditions. These results supported the potential of enteric coating to enhance the pharmacokinetic performance of the abiraterone acetate silica–lipid formulation, which was subsequently confirmed in vivo. Silica–lipid systems enhance the bioavailability of poorly soluble drugs by improving solubilisation and maintaining supersaturation; however, their low drug loading often necessitates large amounts of carrier material, constraining clinical translation. By preserving formulation integrity and enhancing bioavailability, enteric coating can mitigate this limitation by reducing the dose required to achieve therapeutic drug levels, thereby lowering the overall formulation quantity and supporting clinical applicability. Collectively, these findings underscore the inherently iterative nature of nanomedicine formulation optimisation [45], whereby adaptive adjustments are made in response to experimental outcomes.

## 4. Conclusions

Lipid-based formulations and silica nanoparticles can improve the solubilisation of poorly water-soluble compounds. However, for weakly basic drugs such as abiraterone acetate, early release under acidic gastric conditions may lead to precipitation and crystallisation upon transition to the more neutral intestinal environment, thereby compromising the biopharmaceutical performance of the enabling formulation. This study demonstrated that restricting gastric release of abiraterone acetate from a silica–lipid-based formulation enhances both in vitro solubilisation and in vivo pharmacokinetic outcomes. In an in vitro lipolysis model, delaying release until the intestinal phase reduced precipitation and increased drug levels in the aqueous phase by more than 50% compared with two-stage gastrointestinal digestion. In Sprague Dawley rats, the enteric-coated formulation achieved a 2.6-fold higher systemic exposure than the non-coated formulation. These findings support the application of enteric coating to lipid-based formulations and silica nanoparticles containing poorly soluble, weakly basic drugs as a strategy to preserve formulation integrity and delay drug release until reaching the primary absorption site in the small intestine.

## Figures and Tables

**Table 1:** Pharmacokinetic parameters of abiraterone following oral administration of abiraterone acetate formulations in fasted male Sprague-Dawley rats.

Parameter	EC-Par-SL 6 mg/kg n = 4	Par-SL 6 mg/kg n = 4	Pure Prodrug ^a^ 25 mg/kg n = 4
**AUC_0–8 h_** (ng·h/mL)	840 (113)	321 (86)	122 (27)
**C_max_** (ng/mL)	178 (46)	88 (59)	47 (9)
**T_max_** (h)	4 [1–6]	3 [1.5, 4]	2 [2, 2]
**F_rel_** (fold)	28.7	11.0	1.0

^a^ Data for pure prodrug are adopted from a previous study [25]. AUC, area under the plasma concentration–time curve; C_max_, maximum observed concentration; F_rel_, bioavailability relative to pure prodrug; T_max_, time to maximum observed concentration. Data are presented as mean (SD) or median [min, max].

## Data Availability

The data presented in this study are available on request from the corresponding author.

## Abbreviations

The following abbreviations are used in this manuscript:

APC	Australian Prostate Cancer
ASDs	Amorphous Solid Dispersions
AUC	Area Under the Curve
BCS	Biopharmaceutics Classification System
C_max_	Maximum Plasma Concentration
CYP17A1	Cytochrome P450 17A1
EC	Enteric Coated
FaSSGF	Fasted State Simulated Gastric Fluid
FaSSIF	Fasted State Simulated Intestinal Fluid
F_rel_	Relative Bioavailability
MADLS	Multi-Angle Dynamic Light Scattering
NHMRC	National Health and Medical Research Council
Par-SL	Parteck Silica–lipid
PDI	Polydispersity Index
SD	Standard Deviation,
Syl-SL	Syloid Silica–lipid
THRF	The Hospital Research Foundation
t_max_	Time to Maximum Plasma Concentration
